# Cardiovascular disease (CVD): assessment, prediction and policy implications

**DOI:** 10.1186/s12889-021-11334-2

**Published:** 2021-07-02

**Authors:** Shazia Rehman, Erum Rehman, Muhammad Ikram, Zhang Jianglin

**Affiliations:** 1 Department of Dermatology, Shenzhen People’s Hospital, The Second Clinical Medical College, Jinan University, The first Affiliated Hospital, Southern University of Science and Technology, Shenzhen, 518020 Guangdong China; 2 Candidate Branch of National Clinical Research Center for Skin Diseases, Shenzhen, 518020 Guangdong China; 3 Department of Biostatistics, School of Public Health, Harbin Medical University, Harbin, China; 4 Department of Mathematics& Statistics, School of Statistics, Southwestern University of Finance and Economics, Chengdu, China; 5 College of Management, Research Institute of Business Analytics and Supply Chain, Management, Shenzhen University, Shenzhen, China

**Keywords:** Cardiovascular disease, CVD, Relative growth rate, Doubling time model, assessment, forecast

## Abstract

**Background:**

The study aims to predict and assess cardiovascular disease (CVD) patterns in highly affected countries such as Pakistan, India, China, Kenya, the USA, and Sweden. The data for CVD deaths was gathered from 2005 to 2019.

**Methods:**

We utilized non-homogenous discrete grey model (NDGM) to predict growth of cardiovascular deaths in selected countries. We take this process a step further by utilizing novel Synthetic Relative Growth Rate (RGR) and Synthetic Doubling Time (Dt) model to assess how many years it takes to reduce the cardiovascular deaths double in numbers.

**Results:**

The results reveal that the USA and China may lead in terms of raising its number of deaths caused by CVDs till 2027. However, doubling time model suggests that USA may require 2.3 years in reducing the cardiovascular deaths.

**Conclusions:**

This study is significant for the policymakers and health practitioners to ensure the execution of CVD prevention measures to overcome the growing burden of CVD deaths.

## Introduction

The upsurge of the Severe Acute Respiratory Syndrome Coronavirus 2 (SARS-CoV-2) infection in early 2020 caused a disease pandemic known as COVID-19 [1, 2]. There is practically no country in the world which is not influenced by COVID-19 and the health services worldwide have never operated under this tremendous burden. Everything begun in Wuhan [3], China, but it has expanded exponentially all over the world. The core of the pandemic then moved to Europe and the US [4]. Studies also shown that patients with Cardiovascular Disease (CVD) are especially at great risk of COVID-19 mortality owing to their frailty and vulnerability [5, 6]. Patients with pre-existent CV risk factors and CVDs are among the most vulnerable with significantly greater risk of developing SARS-CoV-2 infection, acquiring CV complications associated with COVID-19, and having undesirable consequences [7].

Since the number of deaths has been increasing globally due to different reasons, CVD is one of the world’s most significant causes of mortality and morbidity. During the past decade, the number of deaths from CVD has increased by 12.5% globally [8, 9]. There are various reasons behind this progressive number of cardiovascular deaths. In 2016, the primarily cause of total global burden of CVD was ischemic heart disease (IHD) contributed to 49% of total burden of CVD, followed by stroke with 33% of total CVD burden. In comparison, other CVD causes account for a significantly lower percentage of the global disease burden [10].

CVDs have affected all the income level countries, especially low- and middle-income countries (LMIC) contribute the greatest portion to the overall CVD burden, specifically in terms of deaths at younger ages than in high-income countries, because of scarce human and financial resources [11–13]. Whereas, several of the largest LMIC had an increase in the overall burden of CVD; in decreasing pattern of percent burden increase, those included: India (15.4%), Bangladesh (27.4%), Indonesia (8.8%), China (6.6%), the Philippine (25.3%), and Mexico (19.7%) [14]. However, the CVD burden remains the most significant per capita in Eastern Europe and Central Asia. While, East Asia and South Asia are roots to the increased CVD burden, because of their growing and aging of the populations [15]. The 2016 Global Burden of Diseased Disease (GBD) report reveals that non-communicable diseases (NCDs) comprise 40% of the total age standardized global disease burden for women and about 50% of the global standardized men ‘s overall age burden. CVD alone accounted for 20% of women ‘s total burden and 24% of men ‘s total burden [16]. The frequency of steady or growing CVD Disability Adjusted Life-Year (DALY) epidemiological drivers vary across different countries. In countries such as Japan, a reduced CVD mortality rate has been offset by accelerated population aging. A slight rise of the CVD rate is associated with population ageing and limited resources in South Asian countries such as Pakistan, India, and Bangladesh [17].

Several epidemiological studies have forecast future trends in the occurrence of CVD and mortality rates for all age groups in various countries of the world [18–20], yet little is known about the absolute burden of deaths. To prepare for future cardiovascular care needs with certainty, the purpose of this study is to forecast the number of deaths caused by CVD from 2020 to 2027 of six higly effected countries which include Pakistan, India, China, Keyna, the USA and Sweden. We employed advanced mathematical modeling, namely Non-Homogenous Discrete Grey Model (NDGM) to predict the cardiovascular deaths of selected countries. The grey forecasting models, especially, the NDGM model, can be superior to other forecasting models in the context of small samples and poor information [21]. Hence, we have utilized NDGM model in the current paper to predict the future trends of CVD of top six countries globally. Further, a novel Synthetic Relative Growth Rate (RGR) and Synthetic Doubling Time (*D*_t_) models employed to undertake a comparative analysis of CVD relative growth rate among six countries. Moreover, Mean Absolute Percentage Error (MAPE) % criterion was used to measure the accuracy of NDGM model. Hence, present research is a pioneer study to forecast relative growth and required time to reduce the number of deaths double in number caused by CVD among six countries. The study is principally significant for the policymakers to convey, empower and stimulate the execution of CVD prevention approaches to end this growing burden of CVD during the ongoing COVID-19 pandemic and time ahead.

The rest of paper follow as: Section 2 represent the research methodology. Whereas the result and discussion section are presented in section 3. Finally, conclusion, policy implications along with study limitations presented in last section is study.

## Research methodology

This section provides steps involved in development of NDGM model. Additionally, synthetic RGR and synthetic D_t_ models have been discussed to analyze the growth and time of CVD deaths for selected countries. Finally, the performance evaluation of NDGM model through MAPE is also elaborated in this section.

### Data source and study population

**Fig. 1:**
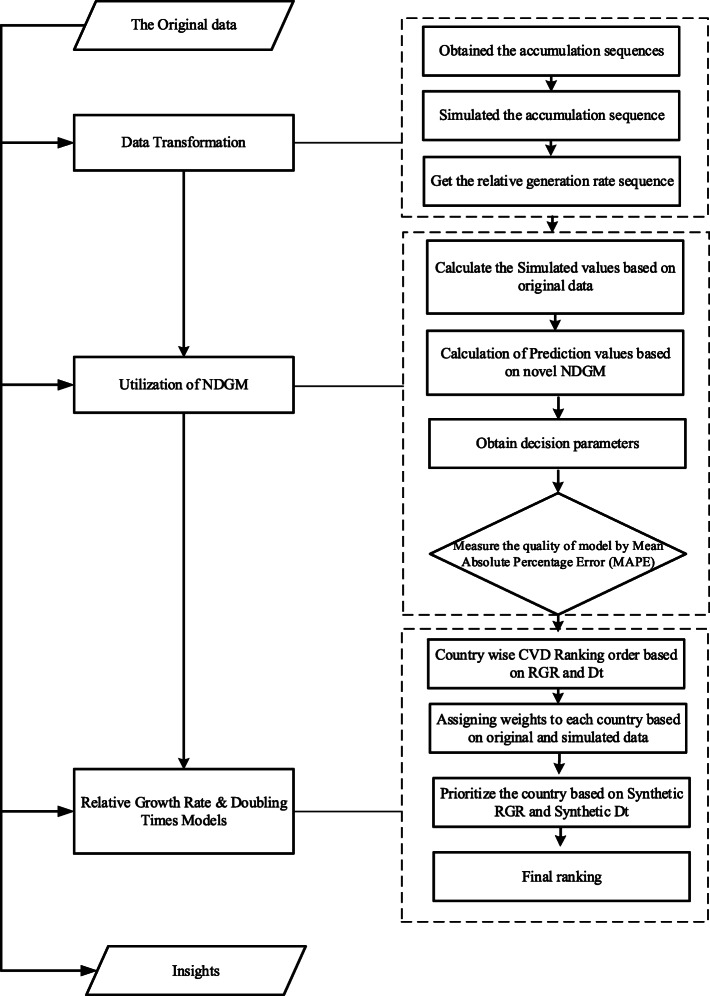
Framework operationalized in this study

The CVD number of deaths data was abstracted from the official website of Our World in Data for the period 2005–2019. The top six countries which are Pakistan, India, China, Kenya, USA and Sweden with highly affected from cardiovascular deaths were selected, globally. The source of the data available at Our World in Data was the global burden of disease (GBD) collaborative network (2016), World Health Organization (WHO) and Institute for health metrics and evaluation (IHME) 2017. Grey system software (v8.0) has been used to forecast cardiovascular deaths for the period of 2020 to 2027 by NDGM. However, MATLAB and MS EXCEL were also used to solve NDGM. The current analysis and modeling methodology were employed for the first time in the study of forecasting CVD related deaths. The structure of forecasting the CVD deaths is operationalized in this study presented in Fig.1.

### Grey forecasting model

In 1982 Deng Julong originally introduced the concept of a Grey Systems Theory (GST) as a scientific theory for predicting uncertain system, which involves limited and inadequate information. Ultimately, theoretical forecasts based on the grey framework surpassed the standards of statistical and mathematical methods [22]. Theory of grey systems has been applied successfully in several fields so far [23–25] and grey prediction theory is a significant branch of the theory of grey systems. There are five major types of grey prediction, such as time series forecasts, calamity forecasts, seasonal calamity forecasts, topological forecasts, and systemic forecasts. Till date, GS theory has introduced a variety of theories and techniques like grey mathematics, grey modeling, grey forecasting, grey clustering, grey decision making, grey programming, grey relational analysis, and grey control, which has been effectively implemented in various fields and has shown satisfactory results [26, 27]. The key benefit of grey theory is that it can manage both with limited and uncertain information with great precision. It functions as an analytical tool particularly in situations where data is inadequate.

#### Nonhomogeneous discrete grey model (NDGM)

In order to predict data, there are different types of grey models, we will analyze NDGM to predict future cardiovascular deaths. The NDGM system is designed on the basis of law of approximation non-homogenous exponential growth in accordance with assumptions of a sequence of real data [24] [28];. Xie et al. [25] recommended that the actual data sequence is a concurrence with a homogenous pattern like GM (1, 1). The accuracy level of NDGM model is considerably improved over other grey models so far as mean sequence value and value set of intervals [29]. NDGM model has been utilized in various fields, for example, in a study the electricity consumption of Turkey was predicted and analyzed the NDGM as best fit and more accurate prediction model over other grey forecasting models [30]. Whereas, Duan et al. forecast the crude oil consumption in China and investigated that NDGM showed superior performance [31].

*x*^(0)^, represent the original data sequence and *x*^(1)^ follows the accumulated data sequence in NDGM model so, we can write as follows:

$$ {\hat{x}}^{(1)}\left(L+1\right)={\beta}_1{\hat{x}}^{(1)}(L)+{\beta}_2L+{\beta}_3 $$

$$ {\hat{x}}^{(1)}(1)={x}^{(1)}(1)+{\beta}_4 $$

Where, ${\hat{x}}^{(1)}$ (L), is the forecasting value of *x*^(1)^ along with parameters *β* 1, *β* 2, *β* 3 and *β* 4. So, we can write the above equation in matrix form as this: if L = 1, 2, and 3 …. n-1

$$ \left[\begin{array}{l}{x}^{(1)}(2)\\ {}{x}^{(2)}(3)\\ {}\kern2em \vdots \\ {}{x}^{(1)}(n)\end{array}\right]=\left[\begin{array}{l}{\beta}_1\\ {}{\beta}_2\\ {}{\beta}_3\end{array}\right]\left[\begin{array}{l}{x}^{(1)}(1)\kern5em 1\kern3.5em 1\\ {}{x}^{(1)}(2)\kern4.5em 2\kern3.5em 2\\ {}\kern0.6em \vdots \kern2.639999em \vdots \kern4em \vdots \\ {}{x}^{(1)}\left(n-1\right)\kern1em L-1\kern2.5em 1\end{array}\right]\kern0.5em $$

The input data shows constant sequence in single case in order to satifsy NDGM parameters β1, β2, β3, and β4 by applying the following relation:

$$ \hat{\beta}={\left({B}^{\mathrm{T}}B\right)}^{-1}{B}^{\mathrm{T}}Y={\left[{\beta}_1,{\beta}_2,{\beta}_3\right]}^{\mathrm{T}} $$

The following formula to be used to calculate β4 for minimizing the sum of square error:

$$ {\beta}_4=\frac{\sum \limits_{L=1}^{n-1}\left[{x}^{(1)}\left(L+1\right)-{\beta_1}^L{x}^{(1)}(1)-{\beta}_2\sum \limits_{j=1}^Lj{\beta_1}^{L-j}\frac{1-{\beta_1}^L}{1-{\beta}_1}.{\beta}_3\right].{\beta_1}^L}{1-\sum \limits_{L=1}^{n-1}{\left({\beta_1}^L\right)}^2\Big)} $$

For further knowledge about NDGM model, its parameter and properties, Liu *et. al.* (2010) is referred [25].

### Performance evaluation approach of NDGM

We employed mean absolute percentage error (MAPE) to evaluate the accuracy of NDGM model. The formula to calculate MAPE % is as follows:

$$ MAPE\left(\%\right)=\frac{1}{n}\sum \limits_{n=1}^k\left|\frac{y^{(0)}(k)-{\hat{y}}^{(0)}(k)}{y^{(0)}(k)}\right|\times 100\% $$

Where *y*^(0)^(*k*) represents the original sequence of data and ${\hat{y}}^{(0)}(k)$ denotes the forecasting sequence data values.

#### Relative growth rate (RGR) and doubling time (D_t_) analysis

To best of our knowledge, there is no model available to check the growth rate for cardiovascular deaths. In this manner, RGR model was used to analyze the relative growth of cardiovascular deaths for selected countries [32]. Two parameters (*D*_*t*_ and RGR) were employed in order to forecast the number of deaths caused by CVD of selected countries by utilizing NDGM model. The equation of RGR is given by,

$$ \mathrm{RGR}=\left(\ln {L}_2-\ln {L}_1\right)/\phantom{\left(\ln {N}_2-\ln {N}_1\right)\left({t}_2-{t}_1\right)}\left({t}_2-{t}_1\right) $$

Where *L*_2_ denotes the cumulative number of cardiovascular deaths in year *t*_2_ & *L*_1_ represent the cumulative number of cardiovascular deaths in year *t*_1_,as in our case, the equation can be reduced to

$$ \mathrm{RGR}=\ln \left({L}_2/\phantom{N_2{N}_1}{L}_1\right) $$

The D_t_ is the time required for publications to reduce the number of cardiovascular deaths for a given RGR is represented as:

$$ {D}_{\mathrm{t}}=\left({t}_2-{t}_1\right)\ln \left[2/\phantom{2\left(\ln {N}_2-\ln {N}_1\right)}\left(\ln {L}_2-\ln {L}_1\right)\right] $$

In our case, it can be written as:

$$ {D}_{\mathrm{t}}=\ln \left[2/\phantom{2\left(\ln {N}_2-\ln {N}_1\right)}\left(\ln {L}_2-\ln {L}_1\right)\right] $$

#### Synthetic RGR and synthetic doubling time model

In any case, if the RGR and *D*_*t*_ make an alternate pattern, when compared with that of actual data pattern create issue, in this regard synthetic Relative Growth Rate (RGR_syn_) and Synthetic Doubling Time (D_syn_) models have been introduced [33]. The equation for Synthetic Relative Growth Rate (RGR_syn_) model can be written as follows:

$$ {\mathrm{RGR}}_{\mathrm{syn}}=\theta \left({\mathrm{RGR}}_{\mathrm{original}}\right)+\left(1-\theta \right)\left({\mathrm{RGR}}_{\mathrm{forecast}}\right) $$

Whereas RGR_original_ denotes the Relative Growth Rate of original data and RGR_forecast_ explains the Relative Growth Rate of predicted values. However, θ indicate relative weights coefficient and its value can be taken as 0.5 in general.

The Synthetic Doubling Time (D_syn_) model is presented as:

$$ {\mathrm{D}}_{\mathrm{syn}}=\theta \left({\mathrm{D}}_{original}\right)+\left(1-\theta \right)\left({D}_{\mathrm{forecast}}\right) $$

Here, D_original_ demonstrates the Doubling Time obtained from original data values, whereas RGR_forecast_ indicate Relative Growth Rate based on forecasting data values.

## Results

We employed NDGM to forecast the relative growth of cardiovascular deaths among six participated countries. The calculated simulated values for the data 2005–2019 are shown in Tables 1, 2, 3, 4, 5, and 6. Table 1 shows the forecasts results for Pakistan. The values obtained from MAPE % demonstrate the effectiveness level of 97.05% which shows NDGM as a best-fit grey model to forecast number of deaths caused by CVD. The simulated values based on NDGM showed an increasing trend for future. Figure 1 has been shown to better understand the comparison between actual data and the simulated NDGM data from 2005 to 2027 against the increasing deaths pattern for CVD in Pakistan.

**Table 1:** Forecasting cardiovascular deaths in Pakistan

Years	Original Data	NDGM	Cumulative	RGR	RGR Mean	D_**t**_	Mean D_**t**_
2005	295,320	295,320	295,320				
2006	298,835	297,535	594,156	0.699	0.223	1.051	2.397
2007	302,305	302,502	896,461	0.411		1.582	
2008	307,782	307,896	1,204,244	0.295		1.913	
2009	313,037	313,753	1,517,282	0.231		2.158	
2010	319,323	320,113	1,836,606	0.191		2.349	
2011	326,241	327,019	2,162,848	0.164		2.504	
2012	334,172	334,517	2,497,021	0.144		2.9	
2013	342,768	342,660	2,839,790	0.129		2.644	
2014	352,846	351,502	3,192,636	0.117		2.838	
2015	362,180	361,102	3,554,816	0.107		2.924	
2016	372,093	371,527	3,926,909	0.100		3.12	
2017	381,421	382,847	4,308,331	0.093		3.072	
2018	395,139	399,214	395,139	0.201		1.914	
2019	408,486	412,578	803,625	0.230		0.351	
2020		422,979	1,226,605				
2021		438,716	1,665,321	0.7521	0.265	1.887	2.125
2022		455,804	2,121,126	0.212		2.112	
2023		474,360	2,595,486	0.252		2.423	
2024		494,508	3,089,995	02174		2.521	
2025		516,386	3,606,381	0.300		2.656	
2026		540,143	4,146,525	0.268		2.721	
2027		565,939	4,712,464	0.228		2.905	
**MAPE %**		**1.56**					

*Abbreviations*: *EGM* even grey model, *DGM* discrete grey model, *NDGM* nonhomogeneous discrete grey model, *RGR* relative growth rate, *Dt* doubling time

**Table 2:** Forecasting cardiovascular deaths in India

Years	Original Data	NDGM	Cumulative	RGR	RGR Mean	D_**t**_	Mean D_**t**_
2005	1,653,599	1,653,599	1,653,599				
2006	1,734,072	1,722,462	3,387,671	0.431	0.224	1.026	2.362
2007	1,818,917	1,822,164	5,206,588	0.312		1.538	
2008	1,909,307	1,918,236	7,115,895	0.257		1.857	
2009	1,996,194	2,010,811	9,112,089	0.217		2.119	
2010	2,095,862	2,100,015	11,207,951	0.169		2.268	
2011	2,197,149	2,185,971	13,405,100	0.157		2.453	
2012	2,285,131	2,268,798	15,690,230	0.14		2.562	
2013	2,351,070	2,348,609	18,041,301	0.126		2.652	
2014	2,418,358	2,425,515	20,459,659	0.115		2.766	
2015	2,490,513	2,499,621	22,950,172	0.107		2.857	
2016	2,583,709	2,571,028	25,533,881	0.038		2.931	
2017	2,632,780	2,639,836	28,166,660	0.271		3.315	
2018	2,706,138	2,712,540	2,706,138	0.19		3.214	
2019	2,770,027	2,790,214	5,476,166	0.617		2.014	
2020		2,831,590	8,307,755				
2021		2,890,911	11,198,666	0.431	0.266	1.825	2.143
2022		2,948,073	14,146,739	0.234		2.217	
2023		3,003,153	17,149,892	0.293		2.342	
2024		3,056,228	20,206,120	0.264		2.522	
2025		3,107,371	23,313,490	0.243		2.608	
2026		3,156,651	26,470,142	0.327		2.777	
2027		3,204,138	29,674,280	0.190		2.862	
**MAPE %**		**2.88%**					

*Abbreviations*: *EGM* even grey model, *DGM* discrete grey model, *NDGM* nonhomogeneous discrete grey model, *RGR* relative growth rate, *Dt* doubling time

**Table 3:** Forecasting cardiovascular deaths in China

Years	Original Data	NDGM	Cumulative	RGR	Mean RGR	D_**t**_	Mean D_**t**_
2005	3,123,852	3,123,852	3,123,852				
2006	3,080,393	3,030,930	6,204,245	0.616	0.236	1.070	2.330
2007	3,126,022	3,171,122	9,330,267	0.408		1.590	
2008	3,260,751	3,308,158	12,591,018	0.352		1.681	
2009	3,440,057	3,442,107	16,031,075	0.242		2.104	
2010	3,610,887	3,573,039	19,641,962	0.263		2.257	
2011	3,744,796	3,701,023	23,386,759	0.165		2.459	
2012	3,844,071	3,826,124	27,230,830	0.161		2.576	
2013	3,872,996	3,948,408	31,103,826	0.153		2.651	
2014	4,039,145	4,067,937	35,142,971	0.141		2.726	
2015	4,221,832	4,184,775	39,364,803	0.133		2.862	
2016	4,344,334	4,298,982	43,709,137	0.105		2.924	
2017	4,377,972	4,410,616	48,087,109	0.095		3.062	
2018	4,519,736	4,532,154	4,519,736	0.299		1.711	
2019	4,626,399	4,678,514	9,146,135	0.168		0.524	
2020		4,730,659	13,876,794				
2021		4,832,572	18,709,366	0.524	0.275	1.240	2.117
2022		4,932,189	23,641,555	0.334		2.146	
2023		5,029,563	28,671,118	0.193		2.315	
2024		5,124,744	33,795,862	0.284		2.451	
2025		5,217,781	39,013,642	0.174		2.514	
2026		5,308,722	44,322,365	0.258		2.612	
2027		5,397,616	49,719,981	0.215		2.701	
**MAPE %**		**4.15%**					

*Abbreviations*: *EGM* even grey model, *DGM* discrete grey model, *NDGM* nonhomogeneous discrete grey model, *RGR* relative growth rate, *Dt* doubling time

**Table 4:** Forecasting cardiovascular deaths in Kenya

Years	Original Data	NDGM	Cumulative	RGR	RGR Mean	D_**t**_	Mean D_**t**_
2005	28,096	28,096	28,096				
2006	28,709	28,780	56,805	0.704	0.212	1.044	2.400
2007	29,283	29,338	86,088	0.416		1.571	
2008	29,907	29,911	115,995	0.258		1.903	
2009	30,578	30,500	146,574	0.224		2.146	
2010	31,216	31,104	177,790	0.183		2.338	
2011	31,891	31,724	209,681	0.155		2.495	
2012	32,452	32,361	242,133	0.143		2.632	
2013	32,895	33,015	275,029	0.127		2.754	
2014	33,453	33,686	308,482	0.117		2.858	
2015	34,203	34,375	342,686	0.105		2.946	
2016	35,107	35,083	377,793	0.068		3.021	
2017	35,992	35,809	413,786	0.041		3.090	
2018	36,555	36,624	36,555	0.255		1.903	
2019	37,321	37,652	73,876	0.324		2.014	
2020		38,107	111,983				
2021		38,914	150,897	0.119	0.261	1.751	2.190
2022		39,742	190,640	0.234		2.146	
2023		40,593	231,234	0.193		2.338	
2024		41,467	272,701	0.165		2.495	
2025		42,363	315,065	0.244		2.628	
2026		43,284	358,349	0.329		2.743	
2027		44,229	402,579	0.206		2.844	
**MAPE %**		**1.9%**					

*Abbreviations*: *EGM* even grey model, *DGM* discrete grey model, *NDGM* nonhomogeneous discrete grey model, *RGR* relative growth rate, *Dt* doubling time

**Table 5:** Forecasting cardiovascular deaths in USA

Years	Original Data	NDGM	Cumulative	RGR	RGR Mean	D_**t**_	Mean D_**t**_
2005	857,472	857,472	857,472				
2006	842,430	817,100	1,699,903	0.684	0.228	1.072	2.514
2007	827,191	817,591	2,527,094	0.528		1.618	
2008	823,970	818,325	3,351,065	0.442		1.958	
2009	814,684	819,425	4,165,749	0.219		2.216	
2010	805,696	821,073	4,971,446	0.166		2.426	
2011	817,311	823,541	5,788,758	0.152		2.546	
2012	821,111	827,238	6,609,869	0.135		2.713	
2013	830,227	832,776	7,440,096	0.128		2.828	
2014	840,356	841,071	8,280,452	0.107		2.928	
2015	857,259	853,496	9,137,711	0.089		3.145	
2016	880,573	872,107	10,018,285	0.052		3.309	
2017	902,270	899,984	10,920,556	0.036		3.414	
2018	941,740	942,407	941,740	0.341		2.045	
2019	1,004,285	101,248	1,946,025	0.215		2.000	
2020		1,097,970	3,043,995				
2021		1,238,298	4,282,293	0.124	0.329	3.412	2.193
2022		1,448,492	5,730,785	0.291		2.104	
2023		1,763,336	7,494,121	0.468		2.302	
2024		2,234,934	9,729,054	0.361		2.431	
2025		2,441,328	12,170,382	0.224		2.554	
2026		2,999,418	15,169,801	0.320		2.856	
2027		3,050,121	18,219,922	0.383		2.935	
**MAPE %**		**1.02%**					

*Abbreviations*: *EGM* even grey model, *DGM* discrete grey model, *NDGM* nonhomogeneous discrete grey model, *RGR* relative growth rate, *Dt* doubling time

**Table 6:** Forecasting cardiovascular deaths in Sweden

Years	Original Data	NDGM	Cumulative	RGR	RGR Mean	D_**t**_	Mean D_**t**_
2005	38,572	38,572	38,572				
2006	38,317	38,716	76,889	0.690	0.208	1.064	2.464
2007	38,053	37,881	114,943	0.402		1.604	
2008	37,525	37,151	152,469	0.283		1.957	
2009	36,792	36,513	189,262	0.216		2.225	
2010	35,644	35,955	224,907	0.173		2.450	
2011	35,642	35,467	260,550	0.147		2.552	
2012	35,070	35,041	295,620	0.126		2.562	
2013	34,559	34,668	330,180	0.111		2.615	
2014	33,961	34,342	364,141	0.098		2.901	
2015	33,768	34,057	397,909	0.089		3.106	
2016	33,710	33,808	431,619	0.081		3.232	
2017	34,163	33,590	465,783	0.076		3.305	
2018	33,399	33,410	33,399	0.286		2.597	
2019	33,233	33,521	66,633	0.102		2.751	
2020		33,087	99,721				
2021		32,960	132,681	0.321	0.254	2.100	2.893
2022		32,849	165,530	0.221		2.765	
2023		32,751	198,282	0.381		2.864	
2024		32,666	230,949	0.253		2.943	
2025		32,592	263,542	02132		3.764	
2026		32,527	296,069	0.116		3.888	
2027		32,470	328,540	0.304		3.978	
**MAPE %**		**2.02%**					

*Abbreviations*: *EGM* even grey model, *DGM* discrete grey model, *NDGM* nonhomogeneous discrete grey model, *RGR* relative growth rate, *Dt* doubling time

With the turn of the century, CVDs became the leading cause of mortality in India. Table-2 represents the results from India with the MAPE accuracy level of 98.35%. The NDGM based simulated values also showed an inclined pattern in progressive number of cardiovascular deaths in future.

Table 3 represents the results for china. The forecast values based on NDGM simulated data showed an anticipated trend until 2027. The MAPE accuracy level turned out to be 96.88%. The NDGM model findings are found consistent with a range of other cardiovascular disease predictions in China, as the burden of CVD was increasing and primary and secondary prevention is likely to be core health policy priorities in the immediate future [34, 35].

**Fig. 2:**
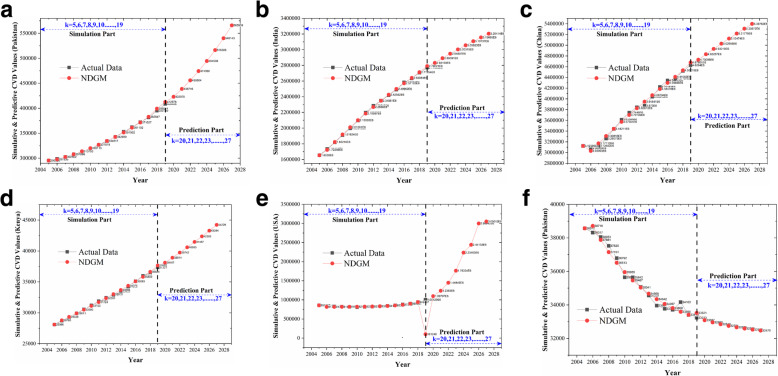
**a** Simulative and Predictive values of NDGM for Pakistan. **b** Simulative and Predictive values of NDGM for India. **c** Simulative and Predictive values of NDGM for China. **d** Simulative and Predictive values of NDGM for Kenya. **e** Simulative and Predictive values of NDGM for USA. **f** Simulative and Predictive values of NDGM for Sweden

Likewise, Tables 4, 5 and 6 represents the results for Kenya, USA and Sweden with MAPE accuracy level of 97.77, 96.8 and 97.74% respectively (Table 8). These findings are similar with some prior findings in which increasing trends have been reported [36]. According to a study in USA if projected trends in ischemic stroke mortality continue, increase in US stroke deaths will outpace overall population growth, with a doubling in deaths by the year 2032 [37]. For an easy and clear comparison of cardiovascular raising trends, Fig. 2a-f can be seen. All participated countries showed an increasing trend in raising their number of cardiovascular deaths, except for Sweden. A study has reported decline in mortality and incidence of cardiovascular related diseases since 1980s in Sweden. Changes in lifestyle have helped to break the increasing trend in CVD [38]. The outcomes reveal that the MAPE accuracy level for NDGM in case of India is found slightly higher than the rest of the five countries whereas Pakistan, Kenya and Sweden showed almost same MAPE accuracy level. The average MAPE accuracy level for NDGM showed a value of 97.44% (Table 8). The anticipated future trends in cardiovascular deaths for the participated countries compel to focus on the importance of increased investment in prevention and treatment of CVD.

### CV death growth and doubling time model based on NDGM

The relative growth rates and doubling time model have been employed to calculate the relative growth of cardiovascular deaths and an expected time to reduce the deaths in selected countries. Table 7 represents the ranking order of six countries for an estimated RGR and doubling time (Dt) as per original and simulated data. The RGR equation of original data showed a ranking order given below:

$$ {\mathrm{China}}_{(0.236)}>{\mathrm{USA}}_{(0.228)}>{\mathrm{India}}_{(0.224)}>{\mathrm{Pakistan}}_{(0.223)}>{\mathrm{Kenya}}_{(0.212)}>{\mathrm{Sweden}}_{(0.208)} $$

**Table 7:** Ranking

Relative Growth rate/ Double Time	Ranking
RGR (original data)	China_(0.236)_ > USA_(0.228)_ > India_(0.224)_ > Pakistan_(0.223)_ > Kenya_(0.212)_ > Sweden_(0.208)_
D_t_ (Original Data)	China_(2.330)_ < India_(2.362)_ < Pakistan_(2.397)_ < Kenya_(2.400)_ < Sweden_(2.464)_ < USA_(2.514)_
RGR (Forecast Data)	USA_(0.329)_ > China_(0.275)_ > Pakistan_(0.269)_ > India_(0.266)_ > Kenya_(0.261)_ > Sweden_(0.254)_
D_t_ (Forecast data)	China_(2.117)_ < Pakistan_(2.125)_ < India_(2.143)_ < Kenya_(2.190)_ < USA_(2.193)_ < Sweden_(2.893)_

**Table 8:** MAPE %

Countries	MAPE % (NDGM)
Pakistan	2.95
India	1.65
China	3.12
Kenya	2.23
USA	3.20
Sweden	2.26
Average MAPE %	2.56
Overall accuracy Level	97.43

To calculate the required time for cardiovascular death reduction among six countries based on original data, the following sequence was observed as per doubling time (D_t_) model:

$$ {\mathrm{China}}_{(2.330)}<{\mathrm{India}}_{(2.362)}<{\mathrm{Pakistan}}_{(2.397)}<{\mathrm{Kenya}}_{(2.400)}<{\mathrm{Sweden}}_{(2.464)}<{\mathrm{USA}}_{(2.514)} $$

The above-mentioned findings demonstrate that the relative growth of cardiovascular deaths in China as an upper middle-income country and USA as a high-income country rank higher followed by India, Pakistan, Kenya and Sweden based on original data. On the contrary, the doubling time model suggest that developed countries like USA and Sweden require an additional time and endeavors to reduce cardiovascular deaths double in number than in developing countries (India, Pakistan, China, Kenya). Therefore, the relative growth rate can be a source of competitive edge among developing and developed countries.

Likewise, we utilized NDGM-based simulated data to find out the status of deaths due to CVD for the period from 2020 to 2027. As indicated by RGR sequence, the following results was acquired:

$$ {\mathrm{USA}}_{(0.329)}>{\mathrm{China}}_{(0.275)}>{\mathrm{Pakistan}}_{(0.269)}>{\mathrm{India}}_{(0.266)}>{\mathrm{Kenya}}_{(0.261)}>{\mathrm{Sweden}}_{(0.254)} $$

Approximately same sequence was observed based on the simulated data. For the period 2020–2027, USA and China may endure progressively number of deaths due to CVD in terms of RGR i.e. (3.29%) and (2.75%) respectively followed-by Pakistan, Kenya, India and Sweden.

As per doubling time (D_t_) model, the following pattern of results is obtained:

$$ {\mathrm{China}}_{(2.117)}<{\mathrm{Pakistan}}_{(2.125)}<{\mathrm{India}}_{(2.143)}<{\mathrm{Kenya}}_{(2.190)}<{\mathrm{USA}}_{(2.193)}<{\mathrm{Sweden}}_{(2.893)} $$

Our findings also revealed that USA and Sweden need relatively additional timespan to double reduce the number of cardiovascular deaths, followed by Kenya, India, Pakistan and China.

By using NDGM (based on actual and simulated data), we forecast the number of cardiovascular deaths for the period from 2020 to 2027.

### Synthetic RGR and synthetic DTM for cardiovascular deaths

Presently a query arises here eventually as which country may endure maximum number of deaths by CVD in the long run. Therefore, to respond the query synthetic indices by original and forecasting values have been calculated.

By using synthetic indices, the sequence obtained for RGR is as follows:

$$ {\mathrm{USA}}_{(0.271)}>{\mathrm{China}}_{(0.255)}>{\mathrm{India}}_{(0.251)}>{\mathrm{Pakistan}}_{(0.248)}>{\mathrm{Kenya}}_{(0.246)}>{\mathrm{Sweden}}_{(0.231)} $$

As per synthetic doubling time model D_t,_ we obtained a sequence as follows:

$$ {\mathrm{China}}_{(2.179)}<{\mathrm{India}}_{(2.263)}<{\mathrm{Pakistan}}_{(2.270)}<{\mathrm{Kenya}}_{(2.276)}<{\mathrm{Sweden}}_{(2.297)}<{\mathrm{USA}}_{(2.386)} $$

Both sequences are found almost similar to the sequences obtained against the actual data thus the results are aligned with the actual data and the feasibility of the synthetic models has also been tested successfully.

## Discussion

In spite of significant reductions in incidence and mortality, CVDs are still the greatest reason for death globally, both in terms of health and economic cost. We built up a forecasting framework to estimate the expected number of cardiovascular deaths in Pakistan, India, China, Kenya, the USA and Sweden by utilizing the most accurate methodology of the grey forecasting framework. Results depicted that grey prediction model was effectively applied to forecast the number of deaths caused by CVD for the period from 2020 to 2027. In addition, this study also suggests an expected time to reduce the number of deaths double in numbers using doubling time (D_t_) formula. All six countries showed an increasing trend in forecasting number of deaths due to CVD for the period 2020–2027, except for Sweden. However, the results indicated that USA and China are more likely to suffer from cardiovascular deaths in future followed by Pakistan, Kenya, India and Sweden. Though China found prone to suffer maximum deaths in future, the doubling time (D_t_) suggests less time expected to control and prevent from cardiovascular deaths double in number.

The results confirm that developed countries need relatively more time to reduce the deaths double in numbers whereas, developing countries require less time to do so. Whereas the USA and China may lead in terms of raising its cardiovascular mortality in future. While in case of lower middle-income countries, India and Pakistan are more likely to suffer from cardiovascular deaths in future followed by Kenya and Sweden. Figuratively speaking, a sword of Damocles hangs over the people of the USA and China, indeed most of the entire world, for the near future. Through CVD prevention and the implementation of workable approaches, we are foreseeing a future wherein the CVD epidemic is being tamed.

Statistics from various parts of the world show a large percentage of cerebrovascular and CV associated diseases in patients with COVID-19, which posed several questions about the higher sensitivity of patients with any of these comorbidities to the novel coronavirus, and also the function of CVD in progression and COVID-19 patient prognosis. Patients with Cardiovascular diseases should be monitored by their healthcare professionals with special preventive procedures for COVID-19 infection. The level of severity of both the primary respiratory syndrome and risk of adverse events is elevated in patients associated with COVID-19 and also with pre-existing cardiovascular diseases. Hospitals and health care networks must embrace a comprehensive approach to provide all patients with the best quality treatment, irrespective of their COVID-19 status. This is especially significant for the cardiology community, considering patients with prior symptoms of CVD and risk factors are potentially more likely to experience COVID-19 and encounter undesirable consequences. As the rate of infection rises, several cardiac patients may seek immediate treatment for either COVID-19 associated diseases or common cardiac complications. Most of these patients would need a cardiac intervention while at the same time getting SARS-CoV-2 infection.

COVID-19, triggered by SARS-CoV-2, is a worldwide pandemic emerging in real time. Cardiovascular and associated comorbidities are very common in COVID-19 patients and those are at greater risk of morbidity and mortality. COVID-19 raised an additional constraint on pre-existing CVDs. Findings from COVID-19 infection with a significant number of patients showed that fatality rate was of 10.5% for CVD among 72,314 COVID-19 cases [39]. Studies have revealed that there is a greater risk of mortality among patients hospitalized with COVID-19 due to CVD. Given the growing number of COVID-19 patients other than regular clinical presentations of illness, CVD in COVID-19 infected patients appear to be alarming [40, 41]. CVDs have played an important role in patient outcomes infected from the virus. Thus, careful review and monitoring of CVD in COVID-19 patients are required, from diagnosis to bedside.

**Fig. 3:**
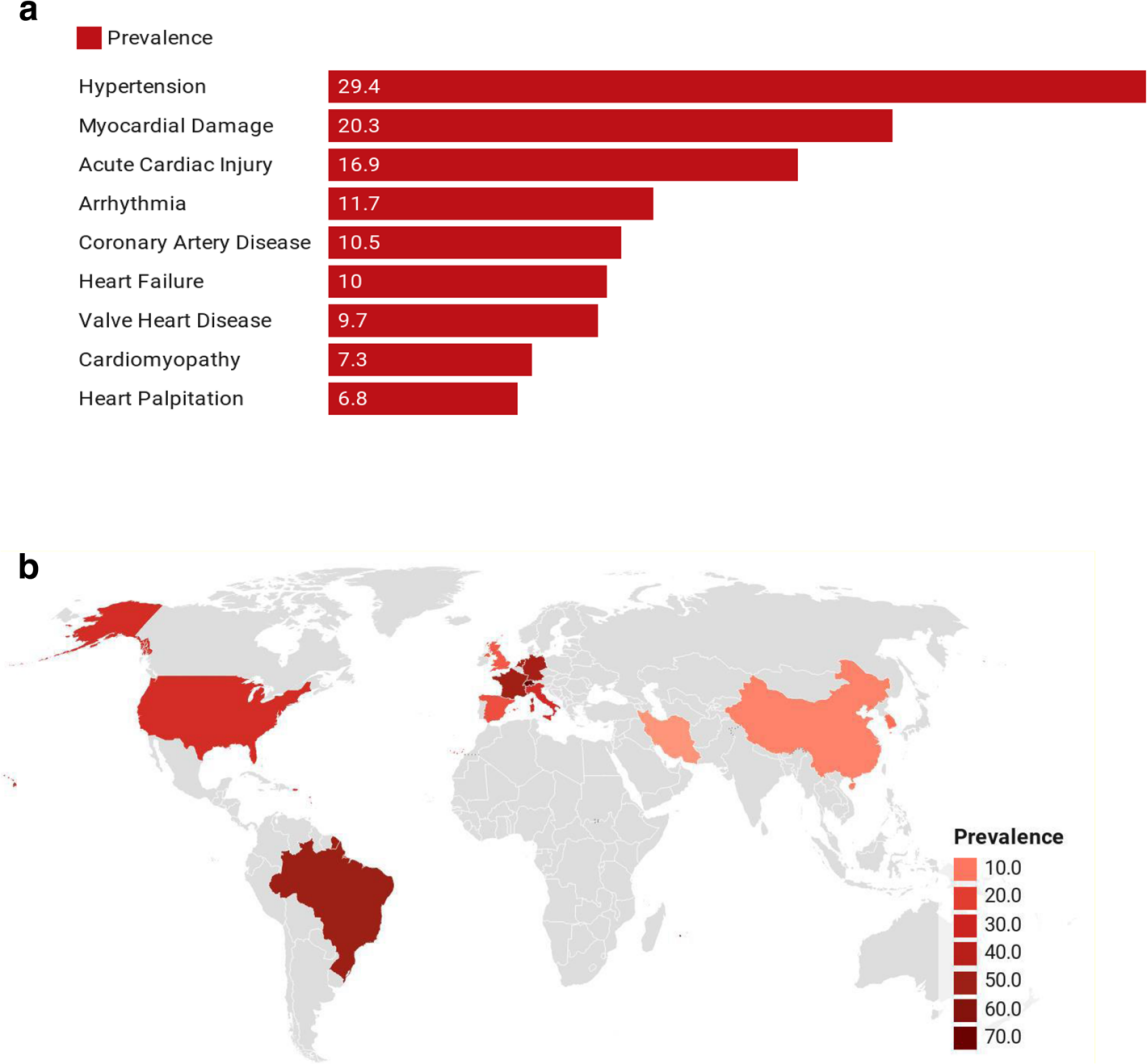
**a** Prevalence of Cardiovascular Complications Among COVID-19 Patients (*Source* [43];). **b** Cardiovascular Disease Burden among COVID-19 Patients in different countries (*Source* [43];)

CV associated complications have been identified in prior respiratory infections with related etiology and their occurrence affects the severity of the illness, so even pneumonia related hospitalization is found related with long-term and short-term risk of CVD [42]. Infection from viruses lead to an imbalance in cardiac supply and demand, as well as an increase in systemic inflammation. Consequently, patients with pre-existing CVD are more likely to experience acute cardiac complications, and thrombosis, and lead to severe infections. An investigation from a meta-analysis analyzed pooled prevalence of CVD in COVID-19 infected patients in various countries. The following are the findings of the meta-analysis: the US (24%), Brazil (50%), Netherlands (44%), Germany (46%), Iran (4%), Italy (25%), China (8%), South Korea (11%), Spain (17%), Switzerland (71%), United Kingdome (15%), and France (48%) Fig. 3a. The prevalence of CV complications in infected patients was shown to be substantially correlated with ICU admissions and mortality in that meta-analysis Fig. 3b [44].

However, valid information to assess the exact burden of CVD is inadequate in many parts of the world, which compels the foundation of nationwide prevention and management policies. It is clear that while many advances have been introduced in our comprehension of worldwide CVD epidemiology, there is wide variation present in data, mainly in MICs and LICs such as Pakistan and India. In these regions, there is a requirement for significantly advanced frameworks for the supervision of risk factors and disease and for strategies that can diminish CVD’s morbidity and mortality at a low cost in ways that are practical and sustainable. The former can be achieved with large but simple health surveys that gather reliable data on health behaviors, CVD associated risk factors, incidence, and mortality, and access to health care in every region of the world.

Though these predictions are sober, they do not need to become true, as CVD is potentially preventable. Improving risk factors at population level in the past obviously had a decisive effect on the reduction in death rates for CVD worldwide. Several studies have shown that there is a considerable decrease in CVD mortality rates among individuals with favorable levels of significant CVD risks [45] [8, 46]. Likewise, people who practice a healthy lifestyle face a comparatively lower risk of suffering from cardiovascular related diseases. A greater emphasis on prevention may therefore alter these anticipated trends in the future and eventually may overcome the growing pandemic of COVID-19.

### Limitations

The present study has several limitations. The data from Our World in Data, however comprehensive and easily accessible to almost all countries and regions, is based on regularly available data from those countries and regions. Variations may occur in the robustness of the data selection and processing and reliability of the cause of the death. Secondly, these predictions are made for developed and developing countries data together, and their generalizability to other countries should be considered with caution. It should be acknowledged that there are many differences between developed and developing countries when comparing health-care systems and how diseases are dealt. We believe that this has kept our analysis conservative. Another limitation of the study is the application of single grey prediction model which is NDGM. The further research can be carried out by employing other grey prediction models and should consider the other regions of the world for more insight information.

## Conclusion

In conclusion, CVD plays a key role in disease burden and mortality in COVID-19 patients. Since CVD complications in COVID-19 patients could be fatal, they must be carefully monitored and managed in the case of an acute illness. It is yet not clear whether the prevalence of cardiovascular comorbidities poses independent risk or if this is affected by other factors such as age etc. The results from the forecasting model against all selected countries showed an increasing trend in terms of raising its number of deaths due to CVD till 2027, except for Sweden. However, the growth rate for the USA (RGR: 2.71%) and China (RGR: 2.55%) was found relatively higher than the rest of the three countries. The findings also revealed that USA (2.3 years) and Sweden (2.2 years) may require relatively additional timespan to double reduce the number of cardiovascular deaths when compared with China (2.1 years). The findings of this study can aid policymakers, doctors, and front-line healthcare workers in making evidence-based decisions and reducing the mortality and morbidity associated with this 21st-century pandemic.

## Data Availability

The data used in the current study is available publicly at www.ourworldindata.com.

## Abbreviations

CVD: Cardiovascular disease
NDGM: Non-homogeneous discrete grey model
EGM: Even Grey Model
DGM: Discrete Grey Model
SARS-CoV-2: Severe acute respiratory syndrome coronavirus 2
RGR: Relative Growth Rate
D_t_: Doubling Time
GST: Grey Systems Theory
IHD: Ischemic Heart Disease
LMICs: Low- and middle-income Countries
GBD: Global Burden of Disease
NCDs: Non-communicable diseases
DALY: Disability adjusted life-years
MAPE: Mean Absolute Percentage Error
WHO: World Health Organization
IHME: Institute of health metrics and evaluation
