# Correction to: Cardiovascular disease (CVD): assessment, prediction and policy implications

**DOI:** 10.1186/s12889-021-11450-z

**Published:** 2021-07-12

**Authors:** Shazia Rehman, Erum Rehman, Muhammad Ikram, Zhang Jianglin

**Affiliations:** 1 Department of Dermatology, Shenzhen People’s Hospital, The Second Clinical Medical College, Jinan University, The first Affiliated Hospital, Southern University of Science and Technology, Shenzhen, 518020 Guangdong China; 2 Candidate Branch of National Clinical Research Center for Skin Diseases, Shenzhen, 518020 Guangdong China; 3 Department of Biostatistics, School of Public Health, Harbin Medical University, Harbin, China; 4 Department of Mathematics& Statistics, School of Statistics, Southwestern University of Finance and Economics, Chengdu, China; 5 College of Management, Research Institute of Business Analytics and Supply Chain, Management, Shenzhen University, Shenzhen, China

**Correction to: BMC Public Health 21, 1299 (2021)**

**https://doi.org/10.1186/s12889-021-11334-2**

It was highlighted that in the original article [1] under the section Relative growth rate (RGR) and doubling time (Dt) analysis, the equations erroneously contained some red text. The original article has been updated.
